# Simultaneous serological assessment of vector and pathogen exposure to support intervention monitoring and understand host–vector interactions

**DOI:** 10.1186/s12936-026-05920-1

**Published:** 2026-04-27

**Authors:** Jessica Bolton, Kevin Kobylinski, Dyna Doum, Allison Tatarsky, Michael Macdonald, David J. McIver, Neil F. Lobo, Elke S. Bergmann-Leitner

**Affiliations:** 1 Immunology Core, Biologics Research and Development, Walter Reed Army Institute of Research, 503 Robert Grant Ave, Silver Spring, MD 20910 USA; 2 Mahidol Oxford Tropical Medicine Research Unit, Bangkok, Thailand; 3 Health Forefront Organization, Phnom Penh, Cambodia; 4 Malaria Elimination Initiative, Institute for Global Health Sciences, University of California, San Francisco, USA; 5 Innovative Vector Control Consortium, Liverpool, UK; 6 Eck Institute for Global Health, University of Notre Dame, Notre Dame, IN USA

**Keywords:** Exposure, Serosurveillance, Vector, *Plasmodium*, Biomarker, Serology, Multiplex, Electrochemiluminescence

## Abstract

Assessing the presence and magnitude of antibodies to pathogens and vector-saliva, *i.e.*, serosurveillance, is a valuable tool to determine actual exposure of individuals to arthropod vectors and vector-borne pathogens under real life conditions. Antibodies to vector saliva proteins have already demonstrated the potential to serve as biomarkers for exposure to mosquitoes. Since various mosquito species can carry pathogens such as *Plasmodia* and flaviviruses, positive serological responses to mosquito saliva have been associated with a higher risk of infections in human populations in endemic areas. A recently developed novel high-throughput multiplex assay simultaneously measures antibodies to vector saliva and pathogen-specific antigens as a tool to monitor the efficacy of countermeasures such as repellants and prophylactic drugs in travelers and residents of African malaria-endemic countries. In the current study, this panel was adapted to include vector-specific probes prevalent in Southeast Asia and test samples from serial cross-sectional surveys in Cambodia. The results revealed distinct immune signatures indicative of distinct patterns of host-vector contact, i.*e.,* bite by infected mosquitoes leading to infection or not, or relapse caused by dormant liver-stage parasites. This approach provides a more detailed understanding of exposure dynamics than traditional surveillance methods, while using a single assay. The study establishes the utility of serosurveillance tools for augmenting conventional entomological and serological methods, particularly in low-transmission settings and when rates of infected vectors are low, thus offering a powerful platform for monitoring exposure to vectors and parasites, disease transmission, and evaluating the efficacy of vector control strategies.

## Introduction

Combating vector-borne diseases requires—apart from the development of new vector control products –an accurate assessments of (a) vector populations, (b) infection rate of vectors with specific pathogens, (c) disease prevalence, and (d) transmission rates to human populations. Researchers and public health authorities use a range of analytical approaches to evaluate these parameters across regions in order to obtain sufficient data to guide and prioritize countermeasure implementation [1]. Until now, researchers have measured changes in transmission potential in the absence of parasites using the concept of vectorial capacity, which describes the ability of a vector population to transmit a pathogen based on parameters such as vector density, biting rate, survival probability, and the extrinsic incubation period. As such, vectorial capacity provides a theoretical estimate of transmission potential independent of parasite presence [1, 2]. However, inaccurate measurements of the components of the formula, especially age-grading, often make this approach impractical [1]. In contrast, field-based measures such as the entomological inoculation rate (EIR)—defined as the number of infectious bites per person over a given time period—capture realized transmission by incorporating both vector dynamics and parasite infection in the mosquito population [3]. While EIR reflects the actual exposure risk, vectorial capacity remains useful for isolating vector-driven components of transmission and for modeling scenarios in which parasite prevalence is unknown or intentionally excluded [4].

Determining the entomological impact of vector control interventions is challenging, particularly in low malaria transmission settings typical of the Greater Mekong Subregion (GMS). In the GMS, it is not possible to use infected mosquitoes as meaningful measure of success of the intervention because the sporozoite rate is typically < 1:100 *Anopheles* [5]. Similarly, epidemiological outcomes are difficult to evaluate in very low transmission settings, especially in the context of randomized controlled trials, as infections become rare, spatially clustered, and sporadic, often occurring in hard-to-reach populations [6]. As a result, very large sample sizes are required to detect differences between intervention arms, reducing feasibility and statistical power. Thus, novel methods to assess the effectiveness of vector control interventions are required in these settings.

Serological surveillance can be used to measure the presence and magnitude of antibodies specific to arthropod saliva and/or pathogens. Serological data have been demonstrated to directly correlate exposure to vectors and the pathogen on both an individual and community level [7, 8]. This insight provides the rationale for the hypothesis that serosurveillance can serve as a complementary tool to conventional surveillance and evaluation methods, particularly for population-level assessments in low-transmission settings. An ELISA-based method to detect responses to vector saliva (gSG6) had been developed for African transmission studies. This gSG6-peptide ELISA has been used extensively in transmission settings across Africa and demonstrated correlation in antibody titers with increasing human landing catch (HLC) numbers, the entomological inoculation rate (EIR), and *Plasmodium* prevalence in humans [8].

A multiplex serosurveillance panel, SeroSignal, was recently developed using the ultra-sensitive Meso Scale Diagnostics (MSD) platform to simultaneously assess exposure to mosquito bites and *Plasmodium* infection [7, 9]. Initial studies demonstrated that the assay can detect even a single exposure to *Plasmodium falciparum*–infected mosquitoes by measuring seroconversion to the mosquito saliva peptide gSG6 from *Anopheles gambiae* alongside antibodies to the sporozoite antigen Circumsporozoite Protein (CSP), while antibodies to erythrocytic antigens such as MSP-1 and AMA-1 serve as markers of recent infection within approximately six weeks [7]. The multiplex approach was previously validated in malaria-endemic regions of Sub-Saharan Africa and among U.S. military personnel deployed to Africa, enabling the identification of biomarkers that distinguish vector exposure from pathogen exposure [9]. In the present study, this panel was expanded for application in Southeast Asia by incorporating saliva proteins from regionally prevalent vectors (*A. dirus*,* A. minimus*,* A. maculatus*) and antigens from *Plasmodium vivax*, the predominant malaria species in the region.

Based on the results from the previous serosurveillance campaign, malaria exposure was defined as immunological evidence of contact with *Plasmodium* sporozoites via mosquito bites, irrespective of whether blood-stage infection occurs. In contrast, malaria infection refers to blood-stage parasitemia detected by conventional diagnostics (PCR). The results from a study on blood samples from U.S. military personnel collected after deployment to African countries with variable malaria burden provided insights into the limitations of current malaria surveillance and diagnostic approaches—based on RDTs and follow-up PCR for febrile illness—compared with serology, which can capture cumulative *Plasmodium* exposure [9]. The SeroSignal panel identified cases in which an infection was likely missed due to inconclusive diagnostic tests leading to under-reporting [9]. When applying the standard diagnostic tests to individuals permanently residing in malaria endemic regions, additional factors interfere with accurate malaria surveillance such as partial immunity to the disease, low density infections, parasites with HRP2/3 deletions, persistence of antigen, and human error (reviewed in [10]. Determining exposure to *Plasmodium* with conventional methods can be obscured when individuals take prophylactic drugs or traditional medicine that prevent or suppress blood-stage infection [11, 12]. However, the mosquito transmission event—even under chemoprevention—can be determined from the immune response to sporozoites (*i.e*., CSP) that is observed with SeroSignal [7, 13]. The results demonstrated that the SeroSignal panel has the potential to fill important gaps in malaria surveillance as it detected *Plasmodium* sporozoite exposures (*e.g.*, transmission) without progression to clinical malaria. In addition, monitoring mosquito saliva antibodies with probes capable of identifying the respective vector, informs on the prevalence and biting rate of specific vectors thus assisting in entomological surveillance [14].

**Fig. 1:**
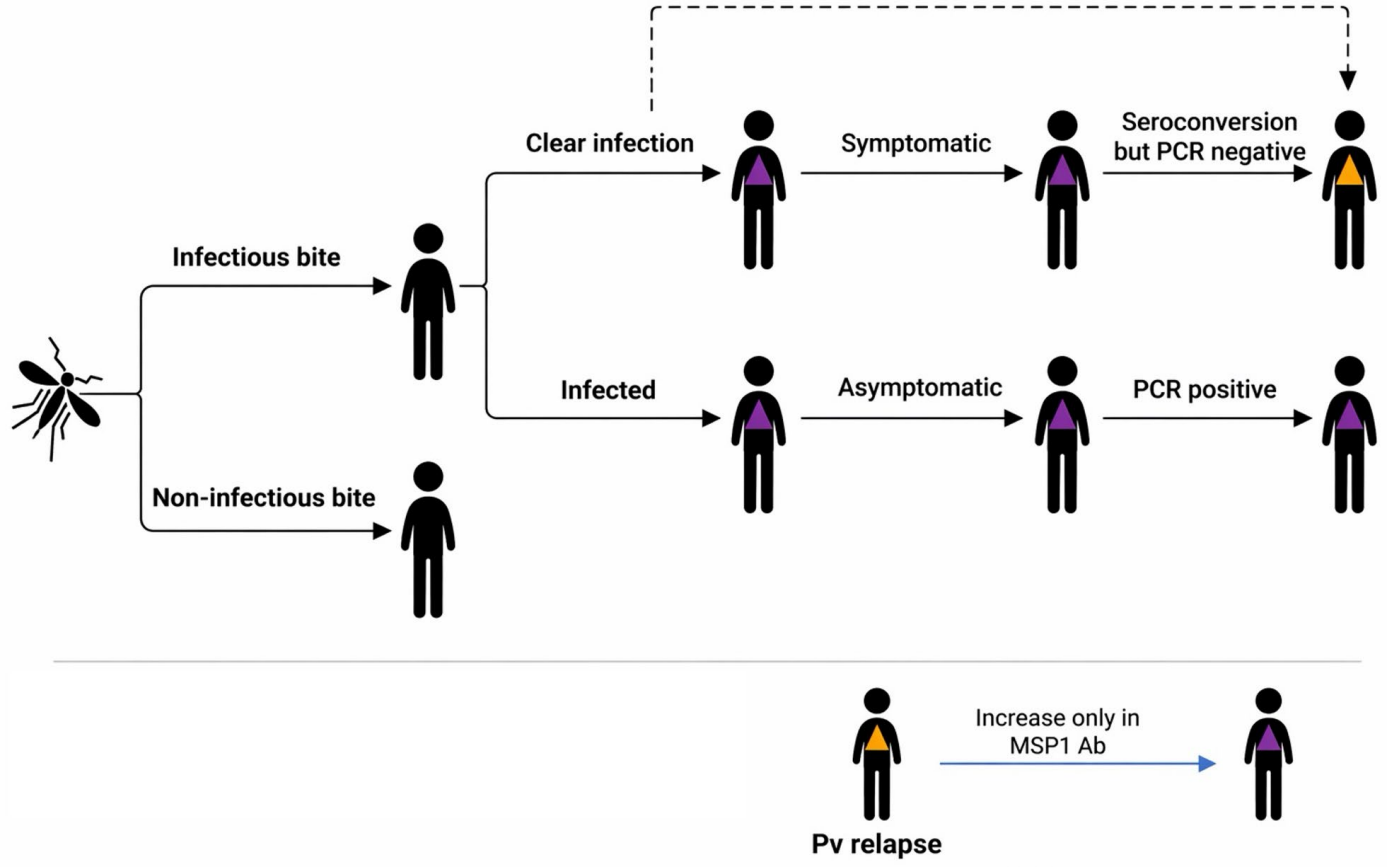
Impact of exposure to vector and Plasmodium on serological profiles. Serological profiles generated by assessing the changes in the levels of antibodies to mosquito saliva, the sporozoite coat protein CSP, and blood-stage antigen MSP1 can inform on exposure (represented by the triangles). The accuracy of the assessment ranges from low confidence (no marked icon), limited (orange marking), to high confidence (purple icon)

The current study aimed to address the link between exposure to uninfected *vs*. *Plasmodium*-infected vectors, *Plasmodium* sporozoites, malaria infection, and associated changes in the serological profile of individuals in Southeast Asia. This analysis was conducted to potentially uncouple infectious mosquito bites from a successful malaria infection. The working hypothesis was based on the fact that the induction of antibodies to CSP may occur even in cases where no malaria infection follows. The following hypothesis was formulated based on these findings (Fig. 1)-when an individual is bitten by a *P. spp*.infected mosquito, there are two potential outcomes:

- (1) Sporozoites injected during the bite are neutralized thus preventing the onset of blood stage infection [15]. Serology should still identify those individuals as the antibody titers to vector saliva and CSP increase leading to seroconversion or a significant increase in antibody titers;
- (2) Bite results in malaria infection thus leads to either symptomatic or asymptomatic disease. In case of asymptomatic disease [16], the blood stage parasite density could be too low to be measured by PCR but still result in seroconversion and/or boost of anti MSP-1 antibodies in serum [17, 18].

To investigate the validity of the working hypothesis, samples were obtained from a recent implementation study of spatial emanators, topical repellants, and etofenprox-treated clothing in Cambodian populations either living or working in forest areas – risk factors for malaria exposure [19, 20]. This study was conducted as part of a larger research program, Project BITE [20]. The samples were used to generate serological profiles with the help of a serosurveillance tool, SeroSignal/Asia. The results of the implementations study itself are detailed in a separate report [21].

## Materials and methods

### Samples/study participants

*Low transmission setting for malaria (Cambodia):* Dried blood spot samples were obtained from a parent study [19, 20] designed to evaluate the implementation of bite prevention tools for malaria in high-risk populations in Cambodia. Forested regions of Cambodia (*e.g*., in areas of Mondulkiri and Kampong Speu provinces) harbor stable vector mosquito populations year-round, even during the dry season, enabling consistent transmission potential when infected humans are present [22]. The BITE project studied the efficacy of new interventions to prevent mosquito bites in protecting individuals living in, or frequenting, the forests [23, 24]. Malaria cross-sectional surveys were conducted in high-risk populations (forest dwellers, forest goers, and forest rangers) at three different time points—T0 (September 2022), T1 (November 2022), and T2 (January 2023), covering the rainy, malaria transmission season (T0 and T1) and reaching into the dry season (T2). Blood samples (n = 6350) collected on filter paper from participants from all target groups were screened for *Plasmodium* species using qPCR. As previously published, the samples set had the following malaria incidence rate [19]: at T0, n = 120 individuals were positive for *P. vivax* (*Pv)*, n = 10 for *P. falciparum* (*Pf)*; at T1, n = 76 individuals were positive for *Pv* and n = 9 for *Pf*; at T2, n = 61 individuals were positive for *Pv* and none were positive for *Pf*. Individuals with complete sample sets, *i.e.*, from all three time points, constituted a longitudinal cohort (n = 332). Antibodies were eluted from dried blood spots as previously described [25]. Briefly, blood samples collected via finger prick were collected on filter paper (Whatman Filter Paper #3) [19]. Small discs (5 mm in diameter) were cut from the filter paper using a handheld metal punch. Two filter paper discs per sample were transferred to a 1.5 mL Eppendorf tube and antibodies were eluted with 100 µL PBS/0.05% (v/v) Tween 20 at 4 °C for 18 h with gentle mixing (rotary shaker at 120 rpm).

*High transmission setting for malaria (Kenya):* This cohort represents a high-exposure African setting (Kombewa, Kenya, baseline immunity prior to vaccination with RTS,S) ([26] and manuscript in preparation) and was included as a comparator to the low-exposure Cambodian study samples. N = 270 samples from adults prior to RTS,S vaccination (n = 128 positive for *Pf* by PCR) were analyzed to illustrate how antibody profiles differ across transmission intensities. We assembled a similar UpSet plot (Fig. 6) using a dataset of serological profiles generated using the SeroSignal panel from Kenyan individuals prior to vaccination [26].

### Antigens for serological testing

Regionally-specific biomarkers of vector- and *Plasmodium* exposure were developed for *An. dirus*, *An. minimus*, *An. maculatus,* and *P. vivax* (Table 1)*.* The SG6 antigen from *An. gambiae* was included as a reference since this protein has been used frequently in sero-epidemiological studies [8]. Markers for *P. falciparum* and their utility for serosurveillance have been described previously [7, 9]. Proteins were obtained from a commercial source (Table 1) and biotinylated using a biotinylation kit (Abcam, Waltham, MA) according to the manufacturer’s instructions. Peptides were synthesized with a N-terminal biotin-tag with a purity of 95% (Atlantic Peptides, Concord, NH).

**Table 1:** Composition of the multiplex serosurveillance panel

Exposure marker	Antigen^a^	Sequence	Source
Vector	*An. gambiae* SG6 ^b^	EKVWVDRDNVYCGHLDCTRVATF	[27]
	*Aedes aegypti* D7	37kD protein (sequence Uniprot P18153)	MyBiosource, San Diego, CA [27]
	*An. maculatus* SG6	EAWRGKLVRGEKYHRLFKKGRQR	[28]
	*An. minimus* SG6	EKVWVDRDRVYCGHIDCTRVATY	[28]
	*An. dirus SG6*	AEPDSECVCPSPRRLPGSAAVPKSVLSSPVKKRAQQ	[28]
Sporozoite	*PfCSP* repeat	(NPNA)_6_	[9]
	PvCSP.R1(VK210) PvCSP.R2 (VK247)	GDRA(A/D)GQPA ANGAGNQPG	[29]
Blood-stage	PfMSP1	MSP1p42	[7]
	PvMSP1	MSP1p42	[7]

^a^ *Pf* *Plasmodium falciparum*, *Pv* *Plasmodium vivax,* *An Anopheles*

^b^ *An. gambiae* SG6 has been used in many other sero-epidemiological studies throughout the world as a universal saliva antigen due to its high cross-reactivities with SG6 proteins from other vector species

### Multiplex immunoassay

The newly developed multiplex electrochemiluminescence immunoassay (ECLIA) methodology is based on the Mesoscale U-PLEX platform utilizing 10-spot ECLIA plates (MSD, Gaithersburg, MD) and performed as previously described [30]. Briefly, biotinylated antigens were diluted to 300 nM using a coating diluent (PBS with 0.5% BSA) and linked with a unique U-plex linker provided by the U-PLEX platform (MSD), vortexed, and incubated at room temperature (RT) for 30 min. The U-PLEX-coupled protein solutions were brought up to 6 mL with Stop Solution, creating a 1 × multiplex coating solution. Plates were coated with the cocktail of proteins and incubated at RT for 1 h on a Titramax plate shaker (Heidolph, Schwabach, Germany), shaking at 700 rpm. Coated plates can be stored for up to seven days at 2–8 °C based on the manufacturer’s information. After incubation, the plates were washed with a working solution of 1 × MSD Wash Buffer (MSD) three times. Sera were diluted to the desired concentration with Diluent 2 (MSD), added to each well and incubated at RT for 1 h on a plate shaker. Plates were washed three times with MSD Wash Buffer and incubated with detection antibody, SULFO-TAG goat anti-human antibody (diluted to 1 µg/mL in Diluent 3 (MSD). Plates were sealed and incubated at RT for 1 h on a plate shaker (700 rpm). After washing, MSD Read Buffer T was added to each well and the plates were read on the MESO QuickPlex SQ 120 (MSD), per the manufacturer’s instructions.

### Statistical analysis

Raw data of the MSD assay are reported as luminescence intensity (MLS). Univariate analysis comparisons between groups (dose cohorts, time points) were made using the Kruskal Wallis test to determine whether any significant differences between groups or time points were present. Posthoc tests were applied as follows: first, the Shapiro–Wilk Normality Test was applied to determine whether a parametric test (Student’s t test) could be applied or a non-parametric test (Wilcoxon signed rank test). P-values were considered significant when p < 0.05. For pairwise longitudinal comparisons across time points, p-values were adjusted using the Bonferroni correction to control for multiple testing.

To visualize longitudinal changes in antibody concentrations, net changes were calculated (by subtracting the amounts at baseline) and the data were transformed using arcsinh (hyperbolic sine) to accommodate negative values [31–33].

### Computational analysis

All statistical analyses was carried out in R (Version 2026.01.0 + 392 (2026.01.0 + 392, [34]) using the *stats*, *glm*, *ggplot2*, *ggpubr, randomforest, caret, lfda, factoextra, UpSetR, venndiagram, mclust*, and *corrplot* packages.

Principal Component Analysis (PCA): PCA was carried out by normalizing and scaling the log-transformed values. Data points were colored by group, and ellipses were generated corresponding to 50% confidence intervals for each group, to identify general trends in the data set.

Correlation plots were generated using pairwise Pearson correlation coefficients calculated from the log-transformed data.

Random Forest Modeling: To verify *P. vivax* PCR positivity based on antibody and serological data, we implemented a Random Forest classifier using the *randomforest* package in R as previously described [35–37]. Briefly, the model was trained on a subset of the data with all predictor variables included. A total of 500 trees were grown (ntree = 500), and at each split, three variables were randomly selected for consideration (mtry = 3). The importance of each predictor variable was assessed using the built-in variable importance metrics. Model performance was evaluated on a held-out test set by predicting both class probabilities and class labels. Performance metrics included overall accuracy, sensitivity, specificity, balanced accuracy, and Cohen’s kappa. Confidence intervals for accuracy were calculated, and a confusion matrix was generated to summarize prediction errors. Out-of-bag (OOB) error estimates from the training set were used as an internal measure of model generalization.

Binary logistic regression: A binary logistic regression was used to determine the probability of *P. vivax* PCR positivity as a function of antibody responses. Predictor variables included gSG6P2, D7, PvCSP.R1, PvCSP.R2, and PvMSP1. Interaction terms between gSG6P2 and D7 and between PvCSP.R1 and PvMSP1 were included to assess potential effect modifications. The model was fitted using the *glm* function in R with a binomial family. Model coefficients were exponentiated to obtain odds ratios (ORs) with 95% confidence intervals (CIs), representing the effect size of each predictor. Model fit was assessed via residual deviance and AIC, and missing observations were excluded from the analysis.

Seroconversion: For some analyses, antibody responses were converted into binary variables (seroconversion: yes/no) using a threshold of z-score > 2 [38–40]. z-scores were calculated as $z=(x-\mu )/\sigma$, where $x$ represents the individual response, $\mu$ and $\sigma$ represent the mean and standard deviation of the entire sample set.

Combinatorial Immune Signatures of Vector and Pathogen Exposure: Binary seroconversion data were used as input to establish immune signatures. To this end, UpSet plots were generated to visualize shared and unique seroconversion patterns among the four exposure markers, *i.e.,* PvPCR-positive (only *P. vivax* infections due to the small sample size of *P. falciparum* infections), mosquito saliva, PvCSP-, and PvMSP1-specific antibodies. The plots were generated with the UpSetR package for R.

## Results

**Fig. 2:**
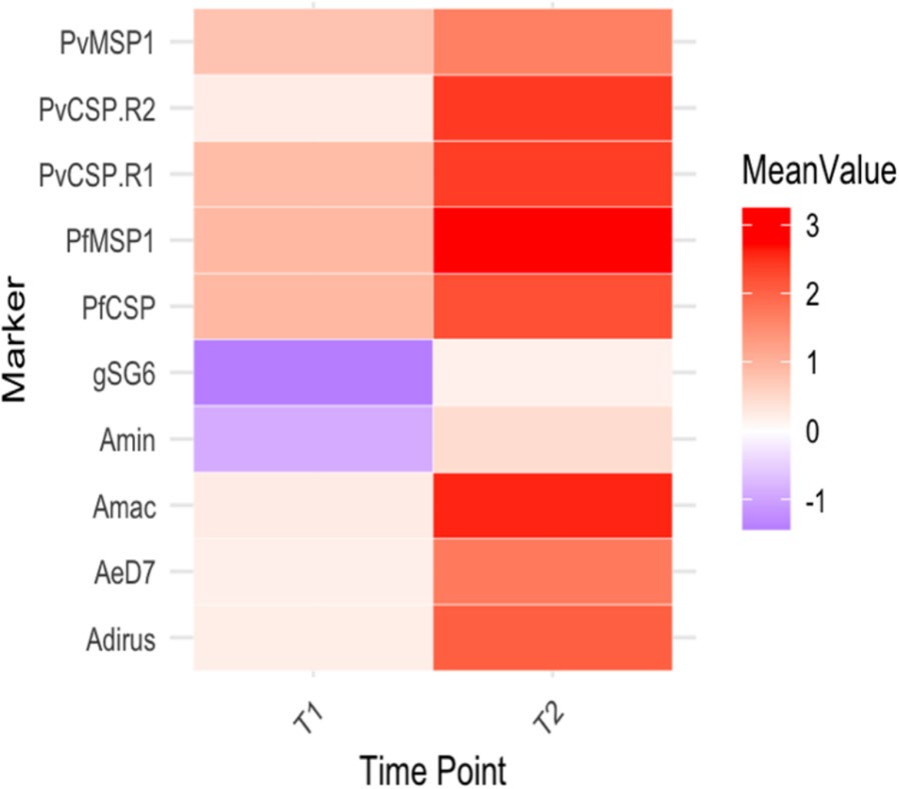
Longitudinal increases in the quantity of antibodies to saliva and Plasmodium-specific antibodies compared to baseline (T0). Data (n = 332, cohort samples from T1 and T2) expressed as the average of net responses (arcsinh transformed, “MeanValue”) to antigenic probes (marker) compared to baseline (T0)

Multiplex serological testing was conducted on all three time points from the longitudinal cohort to establish antibody profiles based on markers of exposure to vectors, *P. falciparum vs*. *P. vivax* sporozoites, and markers of present or recent (< 8 weeks) malaria infection (Table 2). We visualized the longitudinal changes in the magnitude of antibodies to analytes in the SeroSignal panel (Fig. 2).

**Table 2:** Comparison of geometric mean antibody responses (95% CI) to serological analytics across study time points

Analyte	T0	T1	T2	p-value
gSG6P2	2040 (1973, 2109)	1843 (1769, 1921)	2076 (1992, 2165)	< 0.0001
D7	1879 (1811, 1948)	1878 (1801, 1960)	2068 (1986, 2154)	0.002
An.dirus	2482 (2390, 2577)	2524 (2379, 2678)	2775 (2659\, 2896)	< 0.0001^5^
An.mac	1943 (1862, 2027)	1979 (1887, 2075)	2243 (2144, 2346)	< 0.0001
An.min	2131 (2055, 2210)	1986 (1901, 2076)	2171 (2083, 2262)	0.011
PfCSP	3495 (3323, 3675)	3613 (3428, 3807)	3805 (3610, 4010)	0.009
PvCSP1	2355 (2135, 2598)	2463 (2225, 2725)	2667 (2419, 2940)	0.011
PvCSP2	1982 (1897, 2072)	2019 (1915, 2127)	2265 (2160, 2376)	< 0.0001
PfMSP1	1679 (1598, 1764)	1748 (1655, 1846)	2036 (1938, 2138)	< 0.0001
PvMSP1	3284 (3124, 3453)	3408 (3230, 3596)	3521 (3344, 3707)	0.06

Geometric Means (95% CI) of analytes by time point, n = 332/time point. p-values indicate statistical significance in magnitude of responses between time points (Kruskal Wallis test)

Posthoc analyses on paired samples to dissect which time points were associated with statistical differences revealed that levels of antibodies specific for gSG6, *An. minimus*, PvCSP.R2 and Pf/Pv MSP1 were significant for T0 *vs.* T1. The differences between T1 and T2 were significant for antibodies to gSG6, *Aedes* D7, *An. maculatus*, *An. minimus*, PvCSPR2 and Pf/Pv MSP1 (Supplementary Table 1).

### Impact of malaria infection on the serological profile

**Fig. 3:**
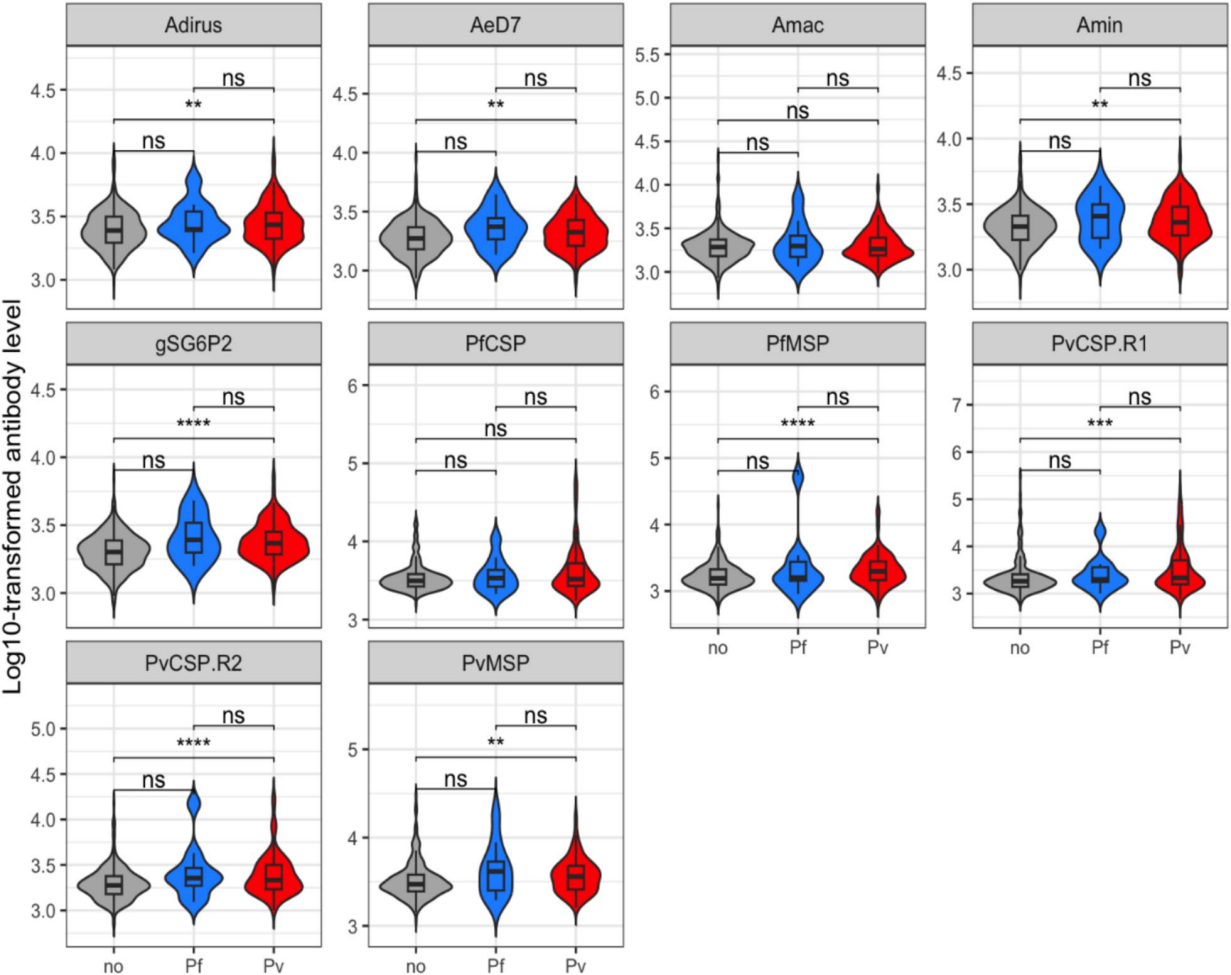
Serological profiles in individuals with and without PCR-confirmed malaria infection. Data are stratified by PCR status: negative for *Plasmodium* blood-stage infection (no, n = 304), P. falciparum positive (Pf, n = 10), and P. vivax positive (Pv, n = 120). Antibody levels are expressed as log10-transformed mean luminescence. Violin plots show the distribution of antibody responses for each group; violin width reflects data density. Internal boxplots indicate the interquartile range with the median shown by a horizontal line, and dots represent individual outliers. Brackets and asterisks indicate statistical significance (Wilcoxon signed rank test:*p < 0.05, **p < 0.01, ***p < 0.001, ****p < 0.0001). Note: Y-axis scales differ between panels

To address the objective, *i.e.*, to determine the impact of exposure to uninfected *vs. Plasmodium*-infected vectors and malaria infection on the serological profile of individuals, the subsequent analysis was restricted to the baseline samples from a trial aiming to assess the efficacy of spatial and topical countermeasures [19, 20]. The serological profile at baseline (T0), stratified by PCR-status, revealed that antibodies to all antigens except for *An. maculatus* saliva and PfCSP were significantly higher in PCR-based malaria positive individuals (Fig. 3).

### Investigating the interplay between Anti-*Plasmodial* and mosquito saliva antibodies

**Fig. 4:**
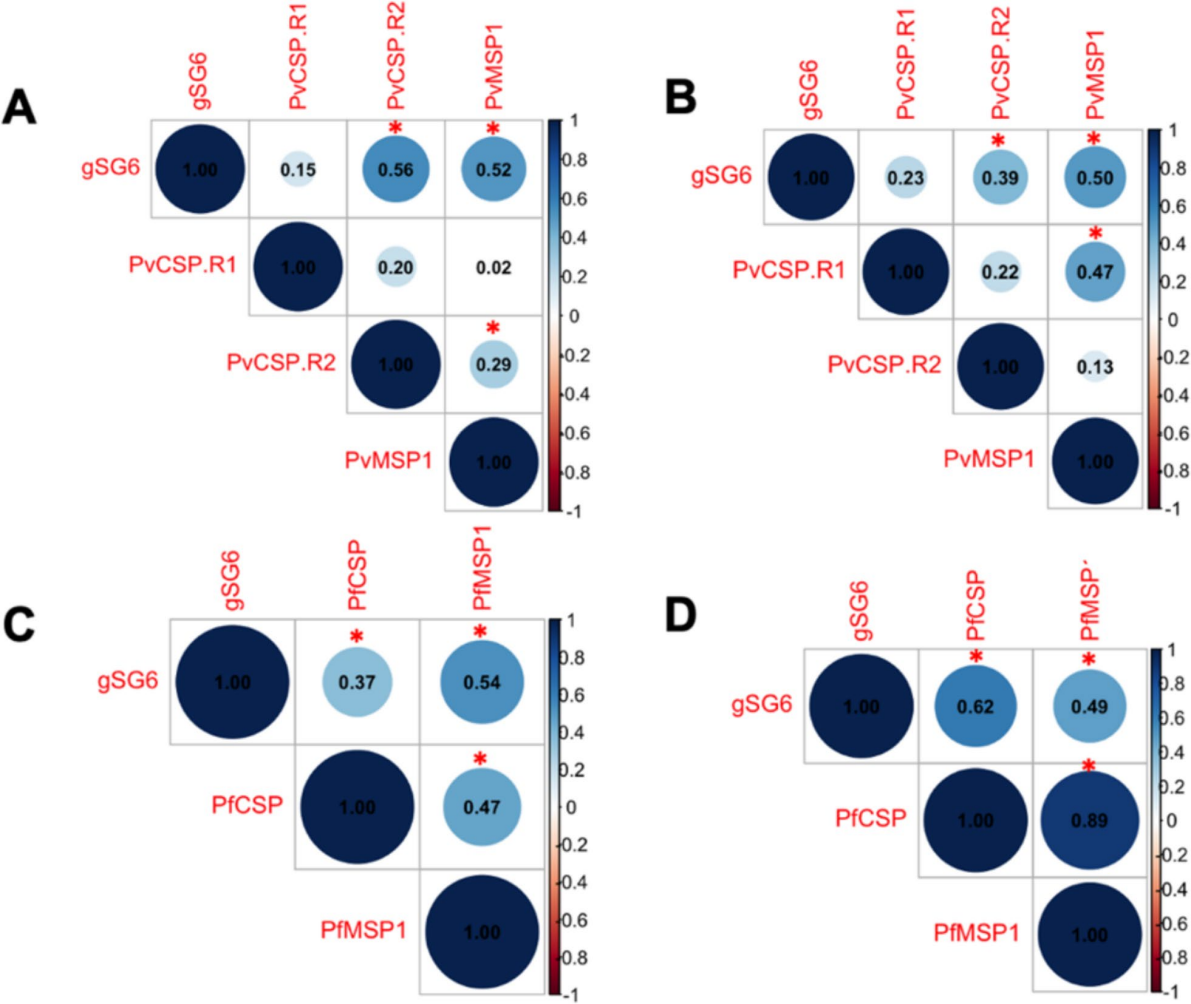
Correlation matrices depict the relationships among CSP-, MSP1-, and mosquito-saliva–specific antibodies in uninfected (Panel **A**, **C**)) and malaria-infected individuals (PvPCR^+^ (Panel **B**) or PfPCR^+^ (Panel **D**)). Dot color and size reflect the strength of the correlation, as indicated by the scale next to each panel. Numbers inside the circles represent the correlation coefficients, and red asterisks denote statistically significant correlations

Next, antibody profiles were examined in individuals who were PCR-negative (PvPCR − and PfPCR −) and in individuals infected with either *P. vivax* or *P. falciparum* (Fig. 3). In individuals with *P. vivax* infection (Fig. 4B), correlation matrices revealed significant associations between antibodies to mosquito saliva (gSG6) and PvCSP.R2 (r = 0.39, p < 0.05) as well as PvMSP1 (r = 0.50, p < 0.01). Interestingly, the correlation between PvCSP.R1 and PvMSP1 was stronger (r = 0.47, p < 0.05) than the correlation between PvCSP.R2 and PvMSP1, potentially indicating more recent exposure to VK210-type parasites (PvCSP.R1). In contrast, individuals who were PCR-negative for *P. vivax* (Fig. 4A) showed a stronger correlation between PvCSP.R2 (VK247 type) and PvMSP1 (r = 0.29, p < 0.05).

Among individuals PCR-positive for P*. falciparum* (Fig. 4D), strong and significant correlations were observed between antibodies specific to mosquito saliva, PfCSP, and PfMSP1 (PfCSP *vs*. gSG6: r = 0.62; PfMSP1 *vs*. gSG6: r = 0.49; PfCSP *vs*. PfMSP1: r = 0.89; all p < 0.01). A similar pattern was observed in individuals who were PCR-negative for *P. falciparum* (Fig. 4C), although correlations were generally weaker (PfCSP *vs*. gSG6: r = 0.37; PfMSP1 *vs*. gSG6: r = 0.54; PfCSP *vs*. PfMSP1: r = 0.47; all p < 0.05). Overall, correlations between CSP- and MSP1-specific antibodies were notably stronger in individuals PCR-positive for *P. falciparum* compared with those PCR-positive for *P. vivax*.

### Identification of serological markers predictive of *Plasmodium* infections

A binary logistic regression was performed to determine the predictive value of the serological profiles. Since the dataset included only a small number of PfPCR + samples, we restricted the regression analysis and modeling to *P.vivax* positive cases. The results identify gSg6 (p < 0.001), PvCSP.R2 and PvCSP.R1 (p < 0.05), and PvMSP1 (p < 0.05) as predicting PCR status. Interestingly, there was a positive correlation between two mosquito saliva proteins, *Anopheles* gSG6 and *Aedes* D7 (p = 0.01) indicative of the co-existence of the two vectors in the geographic area the subjects were residing in. The interaction between gSG6 and D7 indicated a modest negative multiplicative effect (OR 0.94, 95% CI 0.90–0.98, p = 0.007), while the interaction between PvCSP and PvMSP1 was not statistically significant (OR 1.20, 95% CI 0.96–1.50, p = 0.115). In summary, in the multivariable binary logistic regression model, higher anti-gSG6, anti-PvCSP, and anti-PvMSP1 antibody levels were associated with increased odds of *P. vivax* PCR positivity.

To validate these results, random forest modeling was applied which confirmed the weight of these parameters in predicting the responses. Performing random forest modeling revealed that an overall accuracy of 77.9% (95% CI = 70.5%−84.2%) with a high (94.3%) sensitivity for detecting PvPCR + subjects, but poor (15.6%) specificity for the PvPCR-negative individuals, *i.e*., the profiles can identify PvPCR + individuals but fail to predict whether an individual is PCR-negative based on the serology. The importance of the individual parameters was highest for PvCSP (1.08), followed by gSG6 (0.65), and PvMSP1 (0.35) confirming the results of the binary logistic regression. For this analysis, the positive predictive value was 81% and the negative predictive value was 41.7%, respectively.

### Combinatorial immune signatures of vector and pathogen exposure

**Fig. 5:**
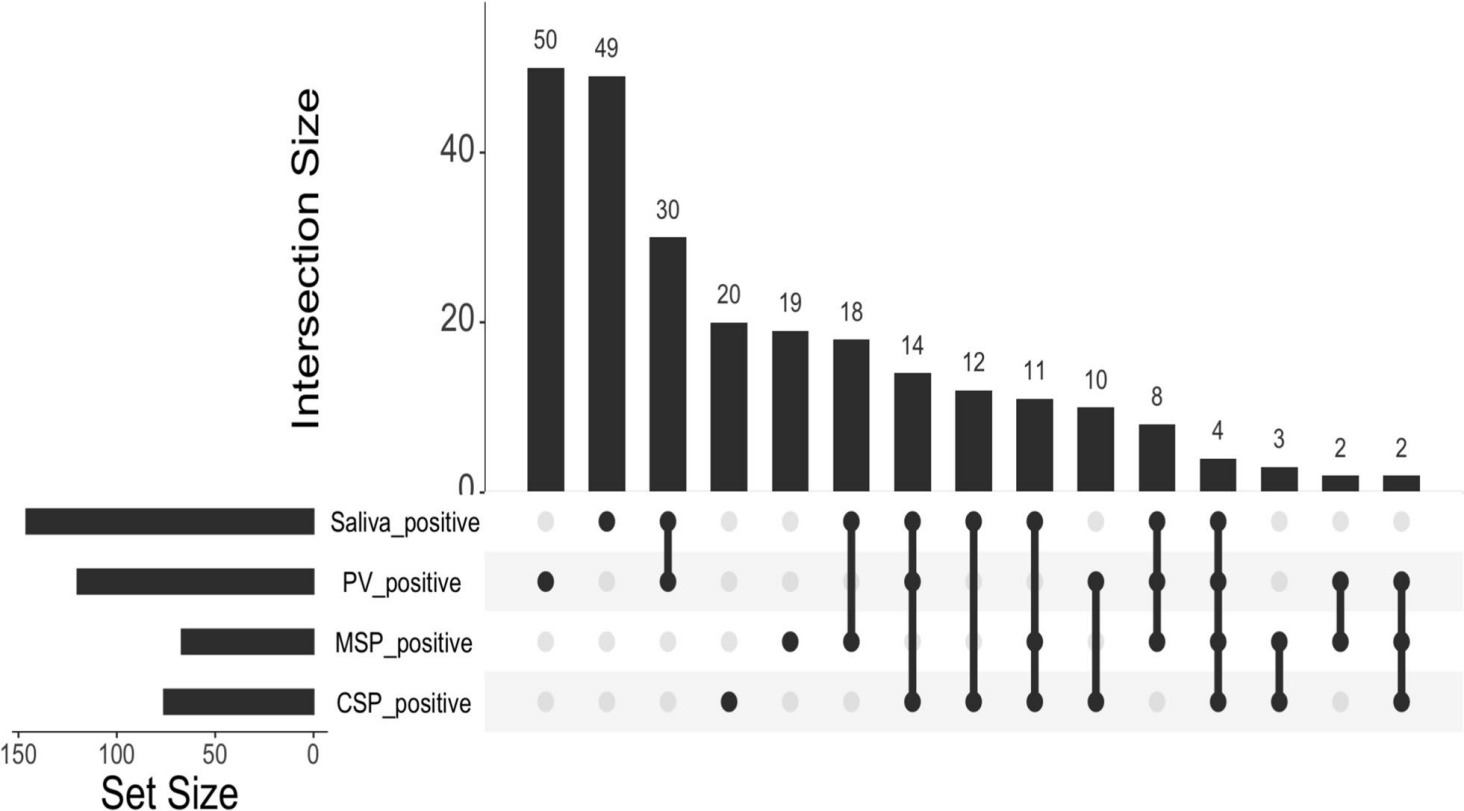
Combinatorial Immune Signatures of Vector and Pathogen Exposure. Seroconversion to each of the test antigens was determined (n = 170 of n = 332 at T0 were converted for at least one antigen). UpSet plot to visualize individual and shared positive responses across parameters, i.e., seroconversion to mosquito saliva, PvMSP1, and PvCSP antigens, and PCR positivity (PV-positive). The horizontal bars on the left indicate the total number of samples positive for each antigen. The vertical bars indicate the size of each intersection, with connected black dots below showing which antigen combinations are included in that intersection. Seroconversion to each antigen is summarized in Supplementary Table S2

By mapping serological profiles for mosquito saliva, PvCSP, PvMSP1, and PCR-confirmed *P. vivax* infection, it became possible—for the first time in this cohort—to see exactly how each exposure translates into seroconversion and potential infection and, thereby, addressing the working hypothesis outlined above. This approach uncovered both shared and unique antibody response patterns, providing a window into the interplay between vector bites and pathogen exposure. Exact seroconversion numbers were reported in Supplementary Table S2 with combinatorial patterns visualized in Fig. 5.

Assessing samples that were only positive for one marker revealed the following profiles: (1) Fifty individuals were PCR^+^ without any seroconversion to Plasmodial or mosquito saliva antigens, (2) 49 samples seroconverted only for gSG6, (3) 20 seroconverted for PvCSP, and (4) 19 seroconverted only for PvMSP1.

Evaluating the serological profile of individuals who were positive for two markers revealed that seroconversion to the profile < mosquito saliva^+^/PCR^+^ > was most frequent (n = 30), followed by < mosquito saliva^+^/MSP1^+^ > (n = 18), < mosquito saliva^+^/CSP^+^ > (n = 12), then < CSP^+^/MSP1^+^ > (n = 3), and least frequently < MSP1^+^/PCR^+^ > (n = 2).

Evaluating the serological profile of individuals who were positive for three markers, the most frequent one was seropositivity for profile < mosquito saliva^+^/CSP^+^/PCR^+^ > (n = 14), followed by < mosquito saliva^+^/CSP^+^/MSP1^+^ > (n = 11), then < mosquito saliva^+^/MSP1^+^/PCR^+^ > (n = 8), and finally < PCR^+^/CSP^+^/MSP1^+^ > (n = 2). Only four individuals were positive for all four markers – < mosquito saliva^+^/PCR^+^/CSP^+^/MSP1^+^ >.

**Fig. 6:**
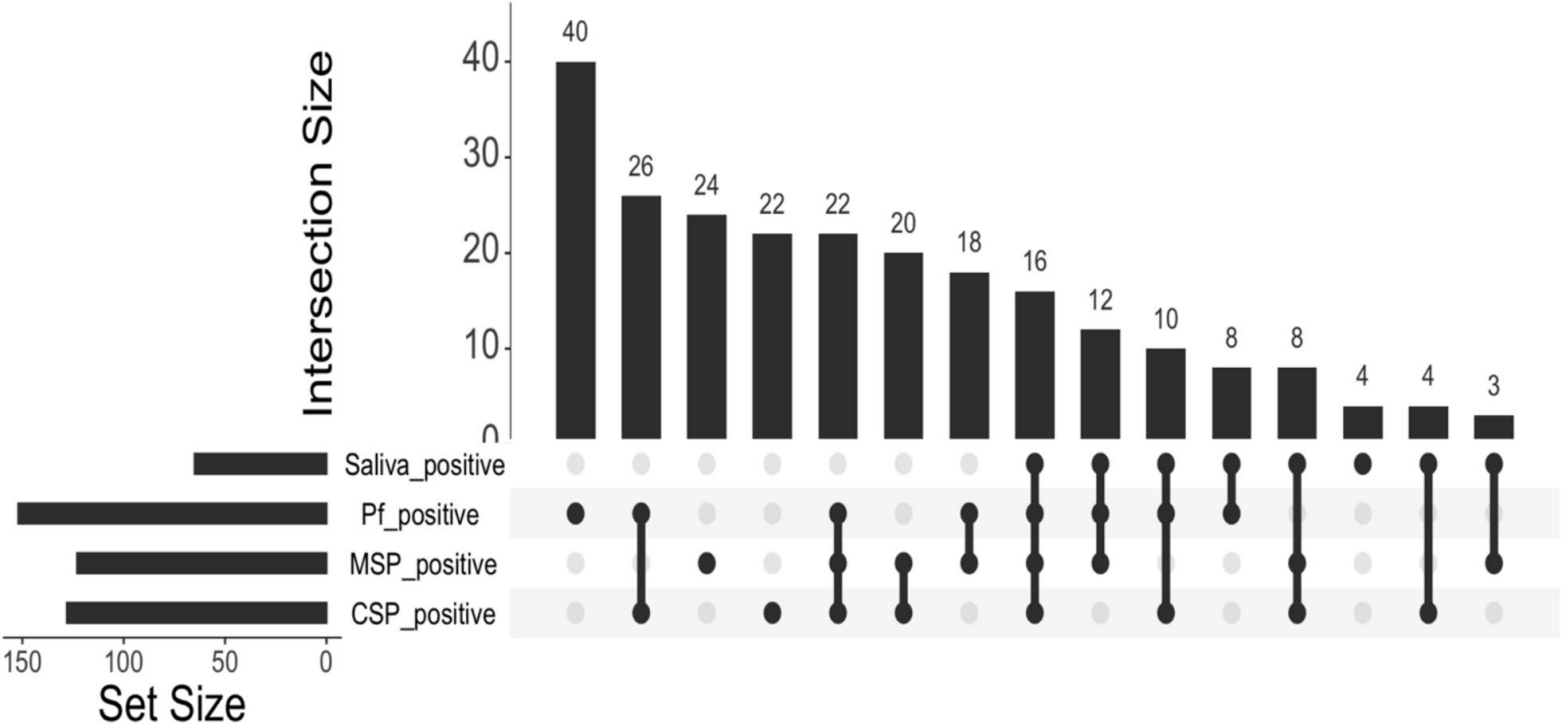
Combinatorial immune signatures of vector and pathogen exposure in malaria-endemic Sub-Saharan region. Seroconversion to each of the test antigens was determined (n = 247 of n = 270 seroconverted for at least one antigen). UpSet plot to visualize individual and shared positive responses across parameters, i.e., seroconversion to mosquito saliva, MSP1, and CSP antigens, and PCR positivity (Pf-positive). The horizontal bars on the left indicate the total number of samples positive for each antigen. The vertical bars indicate the size of each intersection, with connected black dots below showing which antigen combinations are included

To determine whether similar combinatorial antibody signatures emerge in a distinct epidemiological context, *i.e.,* in Africa, the same analysis was performed on a dataset of Kenyan adults [26] where the SeroSignal panel had been used to characterize baseline antibody responses to mosquito saliva and *P. falciparum* antigens (Fig. 6). Most individuals in that cohort were only PfPCR^+^ followed by individuals that were positive for mosquito saliva and PCR. Compared to the data from Cambodia, proportionally far more Kenyan individuals were positive for all four parameters (n = 16 out of n = 237). More individuals (n = 22 of 247) seroconverted for < mosquito saliva^+^/CSP^+^/PCR^+^ >. Mosquito saliva-positivity in Kenyan samples was less frequently part of multi-marker combinations compared to Cambodian adults because of very high background reactivity to saliva markers, indicative of the continuous exposure to large numbers of mosquito bites.

## Discussion

Serological analyses were conducted using the SeroSignal assay to generate complementary data in support of Project BITE (Bite Interruption Toward Elimination) [19, 20], a multi-faceted study aimed at evaluating novel mosquito control strategies in an effort to overcome the final barriers to malaria elimination in Cambodia. The results of the present study confirmed that the SeroSignal surveillance tool provides novel insights into host-vector interactions by simultaneously measuring biomarkers of exposure to vector and pathogen and that the adapted antigen panel is suitable for use in Southeast Asia (Fig. 5).

The results demonstrate that seroconversion to CSP and/or MSP1 does not consistently correlate with PCR-detected malaria infection, highlighting the complex and potentially decoupled dynamics between exposure, infectious inoculum, and the induction or boosting of antibody responses. Several factors may contribute to this observation: First, antibody responses can persist long after parasite clearance, meaning that individuals may remain seropositive even in the absence of detectable parasitemia [29]. Second, low-density infections may fail to trigger a measurable serological response or may be missed by periodic PCR testing [41, 42], introducing temporal mismatches between exposure and detection. Third, heterogeneity in individual immune responses and prior exposure histories can further decouple infection status from seroconversion patterns [43–45]. Nevertheless, serosurveillance remains a powerful tool for capturing cumulative exposure over time, detecting patterns of transmission that may be missed by PCR alone, and identifying population-level risk and hotspots of malaria exposure [46]. This underscores the need for integrated surveillance approaches that combine serological markers of exposure with direct measures of infection in both humans and vectors to more fully capture malaria transmission dynamics in local populations [47].

Serological markers reflective of vector exposure offer a valuable complement to standard diagnostics particularly in low-transmission settings [8, 48, 49] where traditional methods may underestimate true exposure [50–52]. The ability to detect mosquito exposure through serology is particularly valuable in areas co-endemic for pathogens such as Dengue, where no prophylactic drugs are available. In such settings, direct entomological methods such as human landing catches may be ethically complicated or pose unacceptable risk, and, thus, serological monitoring provides a safer alternative for assessing vector exposure. Serological detection of mosquito exposure can help guide in the design of field studies and assess transmission potential without the need to rely on observing clinical outcomes of infections with mosquito-borne pathogen. This is particularly important when considering that, as shown in this study, not all infectious bites result in a successful infection. Moreover, serological data may also be beneficial in low transmission areas where statistically valid studies are hard to conduct due to the need for very large sample sizes. Integrating serological data with entomological and behavioral surveillance data would enhance the panel’s ability to evaluate the impact of vector control and vaccine interventions over time and across multiple phases of the disease transmission cycle thus enabling the understanding of where transmission or protection occurs. This expanded approach will enhance the understanding of host-parasite interactions and support more accurate evaluation of intervention efficacy.

The stratification of serological data by individual PCR status (Fig. 4) demonstrated that antibody responses to vector saliva, pre-erythrocytic CSP (a marker of exposure to *Plasmodium*-infected mosquitoes), and erythrocytic MSP (a marker of blood-stage infection) are strong predictors of PCR-confirmed malaria infection. This association was further supported independently by the results of a binary logistic regression (Table 3) and machine learning analysis using a Random Forest model, in which CSP-specific antibody levels contributed the most accurate classification of infection status. A recent meta-analysis of serosurveillance studies specifically aiming to address the identification of malaria asymptomatic patients based on serology suggested the potential of serosurveillance to detect *P. vivax* exposure even those missed by traditional diagnostics [53]. That review highlights not only the potential of serosurveillance as an actionable tool for malaria control, but also the limitations including the need for standardization and validation across different transmission settings. It was hypothesized that even in low-transmission settings, highly sensitive tests are essential to accurately measure intervention [53]. This is based on reports where serological analyses failed to accurately inform on malaria infections even when employing a multiplex platform [53]. In contrast, in the current study a single serological marker, *i.e.,* CSP, was sufficient to assess exposure to malaria owing to the high sensitivity of the SeroSignal assay. The underlying electro-chemiluminescence platform has several advantages over bead-based flow cytometric assays such as Luminex, particularly a wider dynamic range (3–5 log of magnitude) due to ultrasensitive detection system, and superior intra- and inter-assay variability (> 2% CV) since no fluidics are involved [54]. Lastly, closely related antigens can be tested in the same well without the risk of antigenic competition (interference) [30]. Each well can accommodate up to ten antigens which results in significant sample sparing, increased throughput of testing and, therefore, significant cost savings.

**Table 3:** Summary of binary logistic regression analysis

Predictor	Estimate	SE^a^	OR^b^	95% CI^c^	p-value
gSG6	0.954	0.186	2.60	1.79–3.77	< 0.001
D7	− 0.575	0.176	0.56	0.39–0.79	0.0011
PvCSP.R2	0.100	0.067	1.11	0.97–1.24	0.137
PvCSP.R1	0.133	0.066	1.14	1.01–1.28	0.043
PvMSP1	0.171	0.070	1.19	1.03–1.37	0.015
gSG6:D7^b^	− 0.064	0.024	0.94	0.90–0.98	0.007
PvCSP.R1:PvMSP^b^	0.180	0.114	1.20	0.96–1.50	0.115

^a^ *SE*  standard error

^b^ *OR* odds ratio

^c^ *95% CI*  95% confidence interval

Dissecting the serological profiles led to several conclusions: (a) Not all exposures to infected mosquitoes may result in a blood-stage infection. Figure 5 shows that seropositivity to PvCSP does not consistently correspond with PCR-detected infection or seropositivity to PvMSP1. (b) The assay may have the potential to distinguish *P. vivax* relapses from infections arising from recent sporozoite exposure. As shown in Fig. 5, seropositivity to PvMSP1 and PvPCR-detected infection do not consistently align with PvCSP seropositivity. Furthermore, Fig. 4 highlights the relationship between PfCSP and PfMSP1 antibodies in *P. falciparum* infections (Fig. 4D), where a strong correlation is observed, compared with the notably weaker correlation between PvCSP and PvMSP1 seen in individuals with *P. vivax* infections (Fig. 4B). and (c) Transmission frequency appears to influence combinatorial antibody signatures. Comparison of antibody profiles from the low-transmission setting (Fig. 5) and the high-transmission setting (Fig. 6) suggests that more frequent exposure is associated with broader or stronger antibody responses. While these observations are consistent with the expected impact of transmission intensity, confirmation in longitudinal studies and additional cohorts will be needed to directly establish causality.

A notable difference between the results obtained with the SeroSignal/Africa versus the SeroSignal/Asia assay was the lack of a significant correlation between anti-PvCSP and PvMSP1 antibodies (and correlation between anti-PvMSP and mosquito saliva) in some PvMSP1-positive individuals (Fig. 5). This points to a possible, unique feature of the assay to be able to identify relapsing *P. vivax* infections that arise from dormant liver stages rather than recent sporozoite exposure. To date, this distinction has largely relied on genomic analyses [55], as traditional serological assessments of *P. vivax* exposure—typically based on antibodies to single *Plasmodium* antigens—have shown limited success (reviewed in [53]). However, multiplex serological assays assessing multi-stage antigens hold the promise of ultimately providing the required tool for *P.vivax* elimination [56]. Serological tools capable of identifying recent exposure generally depend on measuring short-lived antibodies [18, 57, 58]. In this regard, the relatively short half-life of vector-specific antibodies offers a major advantage: they enable sensitive detection of recent changes in mosquito exposure and, therefore, provide useful biomarkers for tracking shifts in transmission potential, whether natural or intervention-driven [59, 60]. Although antibodies to CSP and MSP-1 (regardless of *Plasmodium* species) can persist for several months (depending on sensitivity of the assay format) [29], incorporating vector-specific antibody responses alongside parasite antigens generates serological signatures that can help differentiate between new infections and relapses.

The current study has several limitations. First, the SeroSignal assay measures antibody responses to both vector saliva and parasite antigens; as such, these markers reflect cumulative exposure and immune history rather than real-time infection status. Previous work has shown that antibodies following exposure to infected mosquitoes can arise within approximately seven days [7], introducing a lag relative to the timing of infection. Antibody persistence and potential cross-reactivity between related antigens may influence measured responses and complicate the interpretation of species-specific exposure in some individuals. Nonetheless, a recent meta-analysis of 42 surveillance studies reported that seroprevalence was positively associated with entomological inoculation rate and malaria endemicity, supporting the utility of serological markers for population-level transmission assessment [8]. Second, the limited sample size may constrain the generalizability of the observed antibody signatures to other epidemiological settings. Comparing the results to a sample set from a different geographic region (Fig. 6) highlighted the need for future studies across diverse transmission settings. Additional work is needed to refine serological panels, validate them across diverse epidemiological settings, and establish threshold values for interpreting recent versus cumulative exposure.

In conclusion, the present study highlights the potential of multiplex serosurveillance to complement conventional epidemiological and entomological approaches for monitoring malaria transmission and guiding targeted control strategies.

## Data Availability

The data are contained within the manuscript and its Supplementary information. Analysis scripts in R can be made available upon request from the corresponding author.
